# Food Allergies and Ageing

**DOI:** 10.3390/ijms20225580

**Published:** 2019-11-08

**Authors:** Massimo De Martinis, Maria Maddalena Sirufo, Angelo Viscido, Lia Ginaldi

**Affiliations:** 1 Department of Life, Health and Environmental Sciences, University of L’Aquila, 67100 L’Aquila, Italy; maddalena.sirufo@gmail.com (M.M.S.); lia.ginaldi@cc.univaq.it (L.G.); 2 Allergy and Clinical Immunology Unit, AUSL 04 Teramo, Italy; 3 Gastroenterology Unit, Department of Life, Health and Environmental Sciences, University of L’Aquila, 67100 L’Aquila, Italy; angelo.viscido@univaq.it

**Keywords:** food allergy, elderly, aging, hypersensitivity, immunosenescence, gut, allergy, inflammation

## Abstract

All over the world, there is an increase in the overall survival of the population and the number of elderly people. The incidence of allergic reactions is also rising worldwide. Until recently, allergies, and in particular food allergies (FAs), was regarded as a pediatric problem, since some of them start in early childhood and may spontaneously disappear in adulthood. It is being discovered that, on the contrary, these problems are increasingly affecting even the elderly. Along with other diseases that are considered characteristics of advanced age, such as cardiovascular, dysmetabolic, autoimmune, neurodegenerative, and oncological diseases, even FAs are increasingly frequent in the elderly. An FA is a pleiomorphic and multifactorial disease, characterized by an abnormal immune response and an impaired gut barrier function. The elderly exhibit distinct FA phenotypes, and diagnosis is difficult due to frequent co-morbidities and uncertainty in the interpretation of in vitro and in vivo tests. Several factors render the elderly susceptible to FAs, including the physiological changes of aging, a decline in gut barrier function, the skewing of adaptive immunity to a Th2 response, dysregulation of innate immune cells, and age-related changes of gut microbiota. Aging is accompanied by a progressive remodeling of immune system functions, leading to an increased pro-inflammatory status where type 1 cytokines are quantitatively dominant. However, serum Immunoglobulin E (IgE) levels and T helper type 2 (Th2 cytokine production have also been found to be increased in the elderly, suggesting that the type 2 cytokine pattern is not necessarily defective in older age. Dysfunctional dendritic cells in the gut, defects in secretory IgA, and decreased T regulatory function in the elderly also play important roles in FA development. We address herein the main immunologic aspects of aging according to the presence of FAs.

## 1. Introduction

Food allergies (FAs) are becoming a relevant public health concern, affecting over 200 million people worldwide and its prevalence is increasing, mainly in developed countries [1]. FAs are characterized by a wide spectrum of manifestations affecting several organs, ranging from mild to severe and life-threatening reactions [2,3]. The diagnosis of a food allergy is complex because different immunologic mechanisms (IgE-mediated, cell-mediated, or mixed) may play a role. As in most immune-mediated diseases, the variability of clinical expression, as well as its growing prevalence, is determined by genotypic, epigenetic, and environmental factors [4,5,6].

FAs are much more common in children than in adults. Most FAs start in early childhood and usually disappear in adulthood. For this reason, FAs are often considered an almost exclusively pediatric disease. However, although its prevalence is greatest in young children, the occurrence of FA reactions is becoming frequent in the elderly [7,8]. Most studies on epidemiology, immunopathogenesis, and clinical manifestations of FAs have been conducted on children or adolescents rather than in elderly people. However, the demographic distribution of the world population is rapidly changing, with the proportion of older people on the rise and a significant percentage of them haveallergic diseases [9,10]. Parallel to these demographic changes, we can therefore expect that FAs, already increasing in the general population, will also increase in the elderly [11].

It is estimated that the current prevalence of allergic diseases in the elderly reaches 10% but this data is underestimated and also destined to increase [12]. An increasing proportion of children with a FA reaches adulthood and old age, and in some of them, the persistence of the allergic problem occurs. Furthermore, FAs can develop in adulthood and the first symptoms can occur even in elderly subjects [13]. However, to date, there is still not much attention given to FAs in aged people, and symptoms related to FAs, such as vomiting, dyspepsia, diarrhea, pruritus, and skin and respiratory manifestations, often remain undiagnosed in the elderly [14].

Besides the lack of epidemiological data, very little is known about the peculiar immunopathogenetic aspects and the clinical presentation of FAs in old people [15].

The aim of this review is to analyze the pathophysiological mechanisms underlying food allergy in the elderly, emphasizing the most peculiar aspects in this segment of the population, which form the basis of possible intervention measures.

## 2. Immune System Remodeling in the Elderly

The genetic background controls immunity and inflammation, and influences both the aging process and the development of allergies. Several underlying mechanisms of FAs in the elderly are now recognized, the first of which is immunosensecence, i.e., the peculiar age-related remodeling of the immune system. During senescence, both innate and adaptive immune reactions are deeply changed, favoring the development of FAs [16].

In the elderly, there is an imbalance of lymphocyte sub-populations, characterized by a decrease of naive lymphocytes with an accumulation of memory and senescent lymphocytes. Dysfunctions of immune regulatory cells, thymus involution, hematopoietic stem cell malfunctioning, dysregulation of apoptotic processes, a stress response, and mitochondrial function all contribute to the remodeling of the immune system in the elderly [17]. How the balance between the Th1 and Th2 branches is influenced by the aging process is still a controversial matter. Peripheral T cells from aged subjects are activated, exhibiting higher HLA-DR and CD69 expressions, as well as the increased production of inflammatory cytokines, including IL-1β, IL-6, IL-17, IL-31, and TNFα [18]. Immune responses are skewed toward a proallergenic Th2 profile. In particular, the increased IL-4, IL-5, and other Th2 cytokine production observed in aged subjects suggests a Th2 dominance in the elderly [19]. Such immune profile is the main substrate of the allergic reaction [20]. Moreover, certain Th1 cytokines that are increased in the elderly, such as IL-17, may also contribute to the progression of allergic inflammation. The age-related derangement of the cytokine profile may therefore influence the development of FAs in the elderly [21].

Increased inflammatory cytokines and antigen-presenting cell dysfunction contribute to allergic sensitization and inflammation in the aging. Dendritic cells, the mainstarter of the adaptive responses, exhibit altered costimulatory molecule expression in frail elderly subjects, conditioning dysfunctional antigen processing and presentation, which can elicit allergic responses [22].

Concerning effector cells of allergic reactions, most studies report a reduction in eosinophil degranulation in response to IL-5 stimulation and a decreased mast cell function notwithstanding a normal number of mast cells in the tissues [23].

A compromised T helper function and defects of the isotype switching leading to impaired immunologic memory and lower response to vaccines have been observed in the elderly [24]. Conversely, the IgE isotype is less compromised by aging [25]. In particular, immunosenescence does not influence IgE levels in aged patients with atopy, suggesting the persistence of allergy propensity into advanced age [14,26].

## 3. The Mucosal Immune System in Aging

Senescence affects not only the systemic immunity but also the local immune responses, especially on the gastrointestinal mucosa. The induction of mucosal tolerance is of paramount importance in mounting protective responses against new dietary antigens, therefore preventing FAs [3]. The gastrointestinal tract is the largest immunologic system with a relevant amount of lymphocytes that are both scattered and aggregated in lymphatic structures (Peyer’s patches). This gut immune system exerts a key role in FA development, in particular in the elderly, when the thymic function has almost disappeared [27]. Age-related changes affecting the local immune responses contribute variously to the development of FAs. Mucosal tolerance induction is impaired in the elderly, whereas the effector phase of the allergic reaction is substantially maintained [28]. The mechanism of tolerance to food allergens is an active and ongoing process, and the age-related derangement of regulatory functions mediated by interactions of specific cell types promotes allergic sensitization [29,30]. Changes responsible for the breaking of oral tolerance take place in the gut associated lymphoid tissue. The oral tolerance is generally established in childhood and persists even over 65, unless new unknown allergens are introduced. Usually, new dietary protein intake may induce de novo sensitization in the elderly, whereas oral tolerance established in childhood and young age is generally maintained [31].

Inflammaging, the condition of chronic inflammation that drives senescence [32], increases the tight junction permeability through the effects of proinflammatory cytokines [33]. The epithelial cells in the gastrointestinal tract are themselves responsible for both the production of large amounts of cytokines and the reduction of the proteins of the tight junctions and occludens zonula, leading to an increased gut permeability [34]. This decreased barrier effect results in a rupture of the mechanism of tolerance, which predisposes patients to FAs. The presence of inflammatory cytokines, such as IFN-γ, IL-6, and IL-1β, in the gut mucosa is an important factor in this process [35].

The presence of opsonizing secretory IgA antibodies against food antigens is a central mechanism of mucosal immunity by reducing the attachment, penetration, and invasion of antigens across the mucosal wall. Secretory IgA supplied through breastfeeding protect newborns against harmful antigen penetration, leading to transient tolerance/immunity against oral allergens. This mucosal first-line defense mechanism deteriorates with age and orally induced antigen-specific IgA responses weaken [36]. The immunosenescence itself is associated with a significant reduction in IgA levels in the aged mucosa due to the decreased production by B cells and plasma cells [37]. The reduced IgA levels can reflect both an impaired migration of IgA-secreting plasma cells and their numerical reduction [23]. The decreased production of hyaluronic acid and mucus in the elderly also leads to a reduction in the mechanical protection and transport of antibacterial and defensive proteins to the mucosal surface, including IgA. Moreover, differences in the IgA repertoire between young and old subjects have been described, a difference that probably conditions a decreased efficacy of the IgA mediated defenses in the elderly [38]. IgA deficiency in the elderly is related to the development of FAs and intolerances.

## 4. Epithelial Barrier and Digestive Function Impairment

In the elderly, the integrity of the gut epithelial barrier is compromised, contributing to the chronic subclinical inflammatory state. Furthermore, the leaky epithelial barrier promotes Th2-immune responses by allowing allergens to penetrate into tissues where they are processed by dendritic cells and macrophages and presented to T cells [39]. Allergen-exposed epithelial cells produce cytokines, including thymic stromal lymphopoietin, which drive Th2 immune responses [40]. Impaired gut permeability therefore contributes to FA development. Derangement of the intestinal barrier integrity associated with aging may arise after gastroenteric mucosa damage [41]. The decreased digestive capacity of the stomach in the elderly, mainly caused by atrophic gastritis, is an additional risk factor for FAs. Gastric atrophy is frequent in the elderly, and depends on underlying diseases, alcohol abuse, or the chronic consumption of drugs, such as proton pump inhibitors or antacids. Long-term use of glucocorticoids determines a variety of serious side effects, including gastrointestinal effects [42]. Chronic alcohol abuse notoriously enhances the gastric mucosa permeability, induces atrophic gastritis, and decreases the gastric secretory capacity [43,44]. The consequent hypoacidity prevents cleavage of the inactive pro-enzyme pepsinogen and the activation of its protease function in the gastric lumen, thus food proteins remain undigested and transit to the intestine. Such intact food proteins can cross the gut mucosa and enter the blood stream, eliciting the production of IgE antibodies. After a consecutive ingestion of the same food protein, the allergen can crosslink IgE on effector cells, namely mast cells, and trigger the release of mediators, including histamine and leukotrienes, which are the elicitors of local and systemic allergic reactions, whose clinical severity is also partly determined by allergen dosage and integrity [45]. Therefore, a physiologically low gastric pH, by allowing an optimal protein digestibility and preventing the sensitizing and eliciting capacity of the allergen, represents an important protective factor against FAs [14].

Food allergens are mostly structurally stable proteins that usually present a greater risk of causing systemic reactions. When digestion is compromised, labile food proteins can also persist partially undigested along the gastrointestinal tract and become food allergens [46]. The gastric protease propepsin is activated only for pH values below 3.0. Furthermore, only acidic chymus entering the duodenum can induce the release of pancreatic enzymes. Thus, because of the decrease in gastric acidity in the elderly population, protein digestion is compromised and harmless proteins are transformed into potentially dangerous allergens [47]. The therapy with proton pump inhibitors in the elderly, through these mechanisms, could thus facilitate the sensitization to food allergens or lower the trigger threshold of the allergic reaction if a FA is already present [48].

Also, age-related changes in organs and systems different from the gut can exert an important role in the development of FAs in the elderly. The skin is one of the main targets of an allergic reaction to food, as well as an important site of primary sensitization. As a result of chronological and environmental factors, the aged skin is characterized by atrophy and dehydration [49]. The progressive loss of structural integrity leads to an impaired immune response and skin barrier function, increased reactive oxygen species and extracellular matrix component, and vascular impairment [50]. Although T-cell-mediated immunity appears decreased, elderly patients can develop contact dermatitis, as well as sensitize themselves through the skin to food allergens [3].

## 5. Age-Associated Microbial Dysbiosis

In addition to the impaired function of the local immunity and increased gastrointestinal mucosa permeability, age associated alterations of the gut microbiota may also favor FA development in the elderly.

The gut microbiota is a complex ecological system that exerts a central role in several physiological functions, and its composition changes throughout the host’s life. It is sensitive to environmental influences and the host’s diet, and depends on the host’s genetics, gender, and the aging process per se [51].

The system of the secretory IgA plays a critical role not just for the defense against infections but also for the modulation of local immune responses through the maintenance of the intestinal microbiota. Inflammatory processes are associated with dysregulation of the homeostatic interactions between the intestinal microbiota and the aging host [52].

Intestinal microbiota exhibit significant age-associated changes in composition and diversity, as well as in functional features, mainly caused by the immune system remodeling and low-grade chronic inflammation, which respectively characterize immunosenescence and inflammaging [22].

Immunosenescence exerts a key role by modifying the host’s response to microbiota, triggering inflammaging, and shifting Th1 versus Th2 responses, thus favoring tolerance dysruption and allergic reaction development [52].

Antibiotics are among the most commonly used drugs in the elderly and are often used improperly. They influence the microbiome composition and function interfering with immune homeostasis. In the geriatric age, antibiotics can further disturb the composition of the microbiota [53,54]. However, even in the elderly, the intestinal flora can be reconstituted by probiotics, but it is not yet known how this can prevent the development of FAs [55].

## 6. Immune Dysfunctions Due to Nutritional Deficits

Together with the peculiar remodeling of the immune system during senescence, the compromised integrity of epithelial barriers and the sub-clinical chronic inflammatory condition commonly observed in the elderly, a central role in sensitization to food allergens is also played by the lack of micronutrients and vitamins [12].

Micronutrients and antioxidants modulate immune responses and it is suggested that their deficits favor the development of Th2 type responses. For example, deficits of iron, zinc, and vitamin D, which are very common in the elderly, may represent additional risk factors for the onset of allergic reactions during senescence [10].

Zinc is an essential trace element that plays a central role regarding the immune efficiency. Zinc intracellular homeostasis, regulated by metallothioneins and specific transporter proteins, is altered in aging, leading to its decreased availability for immune functions. A reduced zinc level, frequently observed in the elderly, could be responsible for a decreased production of Th1 cytokines, whereas this does not affect the production of Th2 cytokines, thus inducing a cytokine imbalance that promotes the development of allergic diseases [56].

Zinc deficiency contributes to thymic atrophy; immature B cell accumulation; and decreased IgM, Ig-G2a, and IgA subclasses. Stress situations, through pro-inflammatory cytokine production, including IL-6 and TNF-α, are often associated withzinc deficiency. Inflammatory cytokines, permanently increased in the geriatric population, bind zinc ions with a consequent reduced zinc bioavailability and altered immune functions. In particular, decreased levels of zinc induce a reduction of Th1 cytokines, such as IFN-γ, IL-2, and TNF-α, while Th2 cytokines, in particular IL-4, are enhanced. Through this mechanism, zinc deficiency could favor the development of FAs in the elderly [57].

Iron deficiency is also frequent in the elderly [20]. The decreased iron level induces impaired humoral responses, and in particular reduces the production of the IgG4 subclass that physiologically captures the allergens before they can bind to the IgE, thus preventing the activation of effector cells, such as mastocytes and basophils [58].

Several studies suggest that vitamin D deficiency is also very common in the elderly, supporting FA development. Immune dysregulation, in addition to an increased parathyroid hormone level and impaired bone health resulting in enhanced risk of fractures, is a serious consequence of vitamin D deficiency in the elderly [59,60]. The active metabolite of vitamin D, calcitriol, influences T lymphocytes and antigen-presenting cells to induce peripheral tolerance by inhibiting inflammatory responses and promoting regulatory T cells [61]. Vitamin D deficiency is therefore associated with an increased risk of autoimmune and atopic diseases, although the association with IgE levels is not clear [62].

## 7. Clinical Features of FAs in the Elderly

The variable natural history and the complexity of the possible pathogenetic mechanisms, as well as several age-associated factors, make the diagnosis and management of FAs in the elderly difficult. Nutritional abnormalities and vitamin deficiencies, as well as hormonal imbalances and inflammaging, interacting with genetics, may alter the immune responses, leading to FA development. The age-related decline of physiological functions, in addition to the immune system remodeling, which characterizes senescence, contribute to confer peculiar clinical findings to FA in the elderly [23,63].

Furthermore, despite the normal or increased number of mast cells in the skin of aged subjects with an allergy and a sufficient positive response to prick tests with specific allergens, elderly show weaker cutaneous responses and less intense pomfoid reactions to histamine control [64]. Therefore, since the positive reactions to skin test for an allergy could be partially reduced in the elderly, creating possible risk of false negative skin test, a specific IgE search to diagnose FA is commonly used in older patients [65].

Frailty, comorbidity, and multi-drug intake are conditions commonly found in the elderly and must be taken into account in the management of aged people affected by FAs. Immunologic reactions to foods can be confused with symptoms of other common age-related diseases or be masked by the use of various drugs. Consequently, the characteristic symptoms of FAs often go unnoticed and this contributes to underestimating FA prevalence in old age [7]. Dryness and hyperkeratosis, with consequent itching and increased risk of skin infections, are dermatologic manifestations that often mimic and/or mask the symptoms of an allergy. Cutaneous symptoms, such as atopic dermatitis and urticaria, could also represent manifestations of FAs in the elderly [25]. However, in addition to FAs, even drugs and systemic diseases, mainly hematologic and immune dysfunctions, can also induce urticaria in the elderly. Underlying diseases must, therefore, always be suspected, especially when a new diagnosis of chronic urticaria is made in an elderly person [66,67]. Although aged individuals can respond to immunotherapy for a respiratory allergy, as well as to biological drugs commonly used for the treatment of allergic manifestations, such as urticaria and atopic dermatitis, they are usually excluded from these kinds of therapy [68,69]. This is due to the frequent presence of age-related clinical conditions that are considered contraindications. Moreover, the common occurrence of comorbidities and multidrug intake can affect the therapeutic response and promote the onset of side effects. However, immunotherapy and biologic drugs could also significantly improve the quality of life in the elderly, reducing symptoms and drug consumption [70].

Anaphylaxis is a severe and life-threatening hypersensitivity reaction that can affect allergic patients at any age [45]. Clinical manifestations of anaphylaxis caused by food allergens are less frequent in the elderly compared to young subjects [71]. However, although less common, anaphylaxis exhibits a worst prognosis in older patients [72]. The anaphylaxis mediators released by the mast cells after the binding of the allergen to the IgE anchored to their surface induce profound functional modifications on the cardiocirculatory system, including vasospasms of coronary arteries with reduced myocardial blood flow and arhythmias [10,73]. The age-related susceptibility of the cardiovascular system to mast-cell-derived mediators and underlying comorbidities, such as coronary diseases, contribute to the increased mortality and frequent cardiovascular involvement during anaphylaxis in aged people [74]. In patients with multimorbidities, multidrug prescriptions are important cofactors complicating anaphylactic events in the elderly [75]. Cardiovascular drugs, increasingly prescribed to the elderly, strongly contribute to thegreater probability of a fatal outcome. Beta-blockers and angiotensin-converting enzyme (ACE) inhibitors, commonly used to treat congestive heart failure and hypertension, may in fact contribute to aggravate the impairment of compensatory mechanisms typical of the elderly [76]. Several other drugs may interfere with allergic effector cells of FAs. Nonsteroidal anti-inflammatory drugs, taken for chronic osteoarticular pain, are relevant cofactors in urticaria and anaphylaxis in aged subjects [67]. Tricyclic antidepressants, monoamine oxidase inhibitor, and neuroleptics may increase the cardiac risk of epinephrine administration. All these different drugs could cause hypotension, accelerate and increase exposure to allergens, and mask the symptoms of a possible allergic reaction. Although the advanced age doesnot represent an absolute contraindication to self-injectable adrenaline prescription in those at higher risk of anaphylaxis, impaired neuro-motor coordination, frequent hypomobility, and the common coexistence of osteo-muscular and arthrosic hand pathologies compromise the ability to use auto-injectors, suggesting caution in this prescription [77].

The intake of antiulcer drugs is common in the elderly to cure gastritis, gastroesophageal reflux, gastric ulcers, or in association withcorticosteroids and non-steroidal painkillers to minimize their gastrolesive effects. Gastric hypoacidity and increased permeability of the upper gastrointestinal tract also occur as a result of therapy with acid-suppressive drugs, facilitating the onset of an FA, as well as eosinophilic esophagitis. Elderly patients treated with proton pump inhibitors or H2-receptor blockers are at higher risk for sensitization because dietary proteins both remain incompletely digested and can cross the mucosal barrier more easily due to the increased permeability, thus becoming allergenic [8,49].

## 8. Conclusions

**Figure 1:**
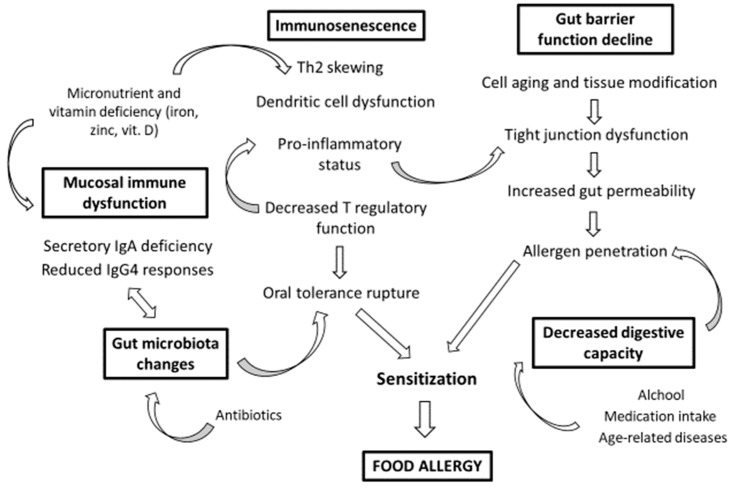
Food allergies in the elderly. The figure shows the main risk factors for the development of a food allergy in the elderly. Immunosenescence and the mucosal immune dysfunction of the gastrointestinal tract are driving forces in the development of food allergies in the elderly. The gut barrier function decline and the compromised digestive properties, as well as the age-related microbiota remodeling, are also central factors for both allergen penetration and sensitization.

Adverse food reactions show peculiar characteristics in the elderly that concern both the pathogenesis and the clinic. FAs in the elderly are driven by immunosenescence, as well as the cell aging and tissue modifications that characterize advanced age. The aged gastrointestinal mucosa is central in the development of FAs in the elderly through its compromised digestive properties and structural changes, as well as the alteration of its immune functions linked to immunosenescence and age-related microbiota remodeling. Among the risk factors for the sensitization to food allergens in the elderly, in addition to chronic damage and inflammation of gut epithelia due to the aging process, there are chronic alcohol consumption, chronic infections, multimorbility, polymedication, and drug side effects (Figure 1).
